# Assessing Proteinuria after Acute Kidney Injury: Is Urine Protein to Creatinine Ratio Sufficient?

**DOI:** 10.34067/KID.0000001103

**Published:** 2026-03-26

**Authors:** Michael Reaume, Edward G. Clark

**Affiliations:** 1 Division of Nephrology, Department of Medicine, University of Ottawa, Ottawa, Ontario, Canada; 2 Kidney Research Centre, Ottawa Hospital Research Institute, Ottawa, Ontario, Canada

**Keywords:** AKI, epidemiology and outcomes, proteinuria

AKI is a common and serious medical condition, affecting up to 20% of hospitalized adult patients worldwide.^1,2^ Key risk factors include older age, underlying health conditions, including pre-existing CKD, as well as cancer, diabetes, hypertension, heart disease, and liver disease, and acute critical illness.^3^ Globally, AKI disproportionately affects patients living in low and middle income countries, where access to preventative care and follow-up, may be limited.^1,2^

AKI is independently associated with all-cause mortality, cardiovascular events, and long-term kidney outcomes, including new-onset CKD and progression to ESKD.^1,2^ Kidney Disease Improving Global Outcomes guidelines recommend assessing kidney function 3 months after hospital discharge for AKI to identify patients at risk of adverse long-term kidney outcomes.^3^ However, Kidney Disease Improving Global Outcomes guidelines do not provide explicit recommendations as to whether clinicians should test for proteinuria using urine albumin to creatinine ratio (ACR) or urine protein to creatinine ratio (PCR).^3^ More recent expert consensus statements favor using urine ACR,^4,5^ which has been shown in epidemiological studies to predict kidney disease progression after a hospitalization with AKI.^6^ However, it is unclear whether urine PCR, which is more readily available in resource limited settings,^7,8^ can serve as a suitable alternative to ACR for predicting long-term kidney outcomes after a hospitalization for AKI.

To address this question, and reported in this issue of *Kidney360*, Kwong *et al*.^9^ conducted a secondary analysis of the Chronic Renal Insufficiency Cohort study, which is a prospective cohort of patients with CKD living in the United States. They identified 554 patients who were hospitalized with AKI and had quantification of proteinuria at a subsequent research study visit within 365 days of hospital discharge. Patients who developed ESKD during the hospitalization were excluded. Over a median follow-up of 2.7 years, 124 patients (22.4%) experienced kidney disease progression, defined as halving of eGFR or development of ESKD. Using Cox proportional hazards models, the authors evaluated the association between doubling of urine PCR and kidney disease progression. In univariable analyses, each doubling of urine PCR was associated with three-fold increased risk of kidney disease progression (hazard ratio, 2.99); the corresponding C-statistic was 0.80, indicating strong discrimination for predicting kidney disease progression. In multivariable analyses that adjusted for age, sex, ethnicity, race, diabetes, body mass index, systolic BP, eGFR, renin-angiotensin system inhibitors use, and AKI severity, the association between doubling of urine PCR and kidney disease progression was attenuated (hazard ratio, 2.01), but the C-statistic increased slightly to 0.86, suggesting even stronger discrimination for predicting kidney disease progression.

This study has several important strengths. The authors leveraged data from a longitudinal, prospective study with detailed patient-level information. Owing to the controlled nature of the research environment, patients attended research study visits at regular intervals, which minimized the possibility of bias related to testing for proteinuria after hospital discharge for AKI. In nonresearch settings, physicians may be more likely to test for proteinuria in patients with other risk factors for kidney disease progression, which can introduce confounding by indication in retrospective cohort studies that rely on administrative data.

However, the study has some limitations. First, the cohort primarily included patients with mild-to-moderate CKD (mean eGFR 43 ml/min per 1.73 m^2^) and mild proteinuria (mean urine PCR was 0.36 g/g) who experienced stage 1 AKI (82%). The authors did not perform statistical tests for effect modification, so it is unclear whether the findings apply equally across all patient subgroups. Doubling of urine PCR may have a stronger association with risk of kidney disease progression in patients with other risk factors for kidney disease progression, such as advanced CKD, diabetes, or severe AKI. Similarly, the results should not be generalized to patients with advanced CKD (*e.g*., eGFR <30), moderate-to-severe proteinuria (*e.g*., urine PCR >1 g/g), or moderate-to-severe AKI (stage 2 or 3) because these patients were underrepresented.

Second, patients were included if urine PCR was measured anytime within 365 days of hospital discharge, which resulted in considerable variability in the time from hospital discharge to urine PCR testing (median, 147 days; interquartile range, 79–233 days). The authors addressed this issue by repeating analyses after stratifying by time for hospital discharge to urine PCR testing (>30 days versus 30–90 days versus 90–180 days versus >180 days). They found comparable C-statistics across these four strata (0.86–0.90), which led them to suggest flexibility in timing of PCR testing. However, this approach did not account for immortal time bias (*i.e*., patients who did not survive long enough to have urine PCR testing were excluded, which means that the cohort of patients with urine PCR testing >180 days from hospital discharge may have represented a healthier subset).

Third, the findings from this study do not tell us whether urine PCR testing after AKI offers prognostic value beyond routine urine PCR testing in CKD without AKI. While the authors adjusted for baseline eGFR and AKI severity, they did not adjust for baseline proteinuria prior to AKI, which is known to be a strong predictor of kidney disease progression in CKD.^10^ Because urine PCR was compared across patients after AKI rather than longitudinally within the same patients (*i.e*., before and after AKI), it remains unclear whether urine PCR after AKI adds prognostic value beyond routine urine PCR testing in CKD.

Finally, the analysis was limited to urine PCR and did not include urine ACR. While urine ACR has previously been shown to predict kidney disease progression after a hospitalization with AKI, the lack of comparable data in the same cohort prevents us from determining whether urine PCR is superior, or at least noninferior, to urine ACR for predicting kidney disease progression among patients with CKD after AKI. Table 1 provides a summary of this study compared with the Assessment, Serial Evaluation, and Subsequent Sequelae-AKI study,^6^ which focused on urine ACR testing.

**Table 1:** Comparison of the Chronic Renal Insufficiency Cohort study (urine protein to creatinine ratio testing after AKI) versus the Assessment, Serial Evaluation, and Subsequent Sequelae-AKI study (urine albumin to creatinine ratio testing after AKI)

Authors	Kwong *et al.* (2025)^9^	Hsu *et al.* (2020)^6^
Dataset	CRIC study	ASSESS-AKI study
Setting	Seven clinical centers and 13 recruitment sites in the United States	Four clinical centers throughout North America
Years of study	2013–2021	2009–2015
Sample size	554	1538
Study design	Prospective cohort	Prospective cohort
Measure of proteinuria	Urine PCR	Urine ACR
Timing of proteinuria measurement	Anytime within 1 yr after discharge from hospital for AKI	3 mo after discharge from hospital for AKI
Outcome	Halving of eGFR or ESKD^a^	Halving of eGFR or ESKD^a^
**Results**		
Univariable	Doubling of urine PCR associated with 2.99 higher risk of kidney disease progression (HR, 2.99; 95% CI, 2.54 to 3.52) C Statistic 0.80	Doubling of urine ACR associated with 1.53 higher risk of kidney disease progression (HR, 1.53; 95% CI, 1.43 to 1.64) C Statistic 0.82
Multivariable	Doubling of urine PCR associated with 2.01 higher risk of kidney disease progression (adjusted^b^ HR, 2.01; 95% CI, 1.62 to 2.48) C Statistic 0.86	Doubling of urine ACR associated with 1.38 higher risk of kidney disease progression (adjusted^c^ HR, 1.38; 95% CI, 1.26 to 1.50) C Statistic 0.85

ACR, albumin to creatinine ratio; ASSESS-AKI, Assessment, Serial Evaluation, and Subsequent Sequelae-AKI; CI, confidence interval; CRIC, Chronic Renal Insufficiency Cohort; HR, hazard ratio; PCR, protein to creatinine ratio.

a Defined as initiation of chronic dialysis or receipt of kidney transplant.

b Adjusted for age, sex, ethnicity/race (Black and Hispanic), diabetes, body mass index, systolic BP, eGFR, and AKI severity.

c Adjusted for age, sex, ethnicity/race (Black and Hispanic), diabetes, body mass index, systolic BP, eGFR, renin-angiotensin system inhibitors use, and AKI severity.

In summary, this well-designed and well-conducted study demonstrated that urine PCR has strong prognostic value for predicting kidney disease progression among patients with CKD who undergo hospitalization with AKI. These findings suggest that urine PCR can be a useful tool to screen for adverse long-term kidney outcomes after a hospitalization with AKI, particularly in resource-limited settings where urine ACR is not readily available.^7,8^

## Supplementary Material

SUPPLEMENTARY MATERIAL
