# Supplementary material for: Assessing Proteinuria after Acute Kidney Injury: Is Urine Protein to Creatinine Ratio Sufficient?
Source: Kidney360. 2026 Mar 26;7(3):464–6. doi: 10.34067/KID.0000001103 (PMC13065210; doi:10.34067/KID.0000001103)
Supplement: SUPPLEMENTARY MATERIAL [file kidney360-7-464-s001.pdf]

ASN American Society of Nephrology | JASN Journal of the American Society of Nephrology | KIDNEY360 Accessing Our World From Every Angle | CJASN Clinical Journal of the American Society of Nephrology

ASN Journal Disclosure Form

As per ASN journal policy, I have disclosed any financial relationships or commitments I have held in the past 36 months as included below. I have listed my Current Employer below to indicate there is a relationship requiring disclosure. If no relationship exists, my Current Employer is not listed.

E. Clark reports the following:
Employer: Nephrology Physicians of Ottawa; Kidney Research Centre - Ottawa Hospital Research Institute; and Advisory or Leadership Role: Editorial Board: Canadian Journal of Kidney Health and Disease.

I understand that the information above will be published within the journal article, if accepted, and that failure to comply and/or to accurately and completely report the potential financial conflicts of interest could lead to the following: 1) Prior to publication, article rejection, or 2) Post-publication, sanctions ranging from, but not limited to, issuing a correction, reporting the inaccurate information to the authors' institution, banning authors from submitting work to ASN journals for varying lengths of time, and/or retraction of the published work.

Name: Edward George Clark
Manuscript ID: K360-2025-001370.
Manuscript Title: Assessing proteinuria after AKI: is urine protein to creatinine ratio sufficient?
Date of Completion: November 19, 2025
Disclosure Updated Date: March 31, 2025

ASN
American Society of Nephrology
JASN
Journal of the American Society of Nephrology
KIDNEY360
Accessing Our World From Every Angle
CJASN
Clinical Journal of the American Society of Nephrology

ASN Journal Disclosure Form

As per ASN journal policy, I have disclosed any financial relationships or commitments I have held in the past 36 months as included below. I have listed my Current Employer below to indicate there is a relationship requiring disclosure. If no relationship exists, my Current Employer is not listed.

M. Reaume reports the following:
Employer: University of Manitoba; University of Ottawa

I understand that the information above will be published within the journal article, if accepted, and that failure to comply and/or to accurately and completely report the potential financial conflicts of interest could lead to the following: 1) Prior to publication, article rejection, or 2) Post-publication, sanctions ranging from, but not limited to, issuing a correction, reporting the inaccurate information to the authors' institution, banning authors from submitting work to ASN journals for varying lengths of time, and/or retraction of the published work.

Name: Michael Reaume
Manuscript ID: K360-2025-001370
Manuscript Title: Assessing proteinuria after AKI: is urine protein to creatinine ratio sufficient?
Date of Completion: November 20, 2025
Disclosure Updated Date: November 20, 2025
